# Low-Temperature Heat Capacity Anomalies in Ordered and Disordered Phases of Normal and Deuterated Thiophene

**DOI:** 10.1021/acs.jpclett.1c00289

**Published:** 2021-02-24

**Authors:** Y. Miyazaki, M. Nakano, A. I. Krivchikov, O. A. Koroyuk, J. F. Gebbia, C. Cazorla, J. Ll. Tamarit

**Affiliations:** † Research Center for Thermal and Entropic Science, Graduate School of Science, Osaka University, 1-1 Machikaneyama, Toyonaka 560-0043, Osaka, Japan; ‡ B. Verkin Institute for Low Temperature Physics and Engineering, National Academy of Sciences Ukraine, 47 Science Avenue, Kharkov 61103, Ukraine; § Grup de Caracterizació de Materials, Departament de Física, EEBE and Barcelona Research Center in Multiscale Science and Engineering, Universitat Politècnica de Catalunya, Eduard Maristany, 10-14, Barcelona 08019, Catalonia, Spain; ∥ Departament de Física, Universitat Politècnica de Catalunya, Campus Nord B4−B5, Barcelona E-08034, Catalonia, Spain

## Abstract

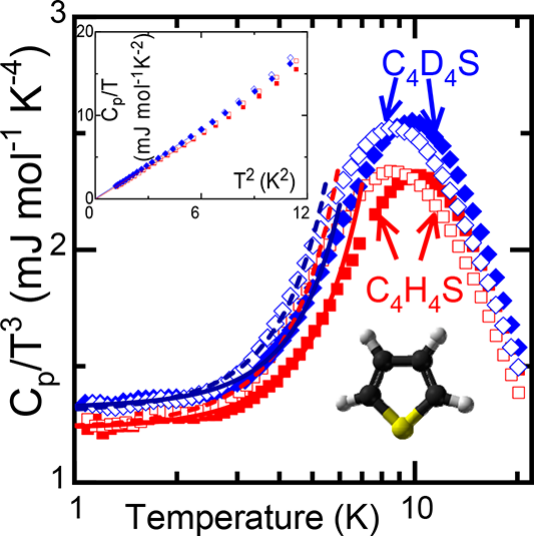

We measured the specific heat *C*_p_ of normal (C_4_H_4_S) and deuterated (C_4_D_4_S) thiophene in the temperature interval of 1 ≤ *T*, K ≤ 25. C_4_H_4_S exhibits a metastable phase II_2_ and a stable phase V, both with frozen orientational disorder (OD), whereas C_4_D_4_S exhibits a metastable phase II_2_, which is analogous to the OD phase II_2_ of C_4_H_4_S and a fully ordered stable phase V. Our measurements demonstrate the existence of a large bump in the heat capacity of both stable and metastable C_4_D_4_S and C_4_H_4_S phases at temperatures of ∼10 K, which significantly departs from the expected Debye temperature behavior of *C*_p_ ≈ *T*^3^. This case study demonstrates that the identified low-temperature *C*_p_ anomaly, typically referred to as a “Boson-peak” in the context of glassy crystals, is not exclusive of disordered materials.

The search for the physical nature of low-temperature anomalies in the thermal properties of glasses and disordered crystals started ∼50 years ago.^1−4^ Yet, to this day both structural and orientational glasses display anomalous properties that are still not fully understood. Among them, the physical origins and conditions of the existence of the so-called Boson peak (BP) stand out,^4−6^ that is, a thermal anomaly leading to a heat capacity (*C*_p_) maximum in the representation *C*_p_/*T*^3^ versus *T*. It is believed that the BP appears as a consequence of the excess of low-frequency states corresponding to a local maximum in the reduced vibrational density of states (*g*(ω)/ω^2^ vs ω), which typically is attributed only to orientational or structural glasses.

The complex dynamics of the glass state is a consequence of low-energy excitations appearing in the vibrational density of states, *g*(ω), which in turn are manifested also in thermodynamic response functions like the specific heat.^7−9^

This letter aims at finding universal behaviors concerning well-ordered crystals, structural glasses, orientational glasses, and glassy crystals obtained by frozen statistical intrinsic disorder. The specific purpose of our study is to evidence some experimental facts that should help in disentangling the physical origin and nature of the BP associated with their heat capacity. With this purpose in mind, we performed highly accurate low-temperature *C*_p_ measurements on normal and deuterated thiophene, since these crystals harbor a unique diversity of fully ordered (FO) and orientationally disordered phases.

Thiophene is a planar heterocyclic aromatic compound (see inset in Figure 1) consisting of a planar five-membered ring with the S atom showing the direction of the molecular dipole. It displays a quasi-fivefold symmetry axis perpendicular to the molecular plane. For both normal and deuterated materials, two similar phase sequences, one stable and one metastable, were broadly characterized, mainly structurally (see Scheme S1 in the Supporting Information). When cooled from the liquid phase (*T*_m_ = 235 K) an orientationally disordered (plastic) orthorhombic (*Cmca* space group) phase forms (I).^10,11^ When further cooled and transitioned through some intermediate phases (II and II_1_), the phase II_2_ is finally formed and remains metastable down to the lowest temperature that was measured (1 K for C_4_D_4_S and 14 K for C_4_H_4_S).^12,13^ Despite both II_1_ and II_2_ phases being metastable, a reversible first-order phase transition at 90.7 K from II_2_ to II_1_ is found when heated. Further heating from phase II_1_ and annealing at ∼160 K results in the formation of the orthorhombic stable phase III (space group *Pnma*). When phase III is cooled, phase IV, an incommensurate superstructure of phase III (*Pbnm* space group), and phase V, a superstructure of phase III corresponding to a doubling of the *a* parameter, are sequentially and reversibly found. With regard to the orientational disorder, phase I displays 20 equiprobable molecular orientations, whereas 10 are exhibited in phases II (as well as II_1_ and II_2_) and III for both normal and deuterated samples. In addition to some subtle differences in the transition temperatures, the main, interesting, and distinguishing experimental difference between the normal and deuterated thiophenes concerns the dynamical disorder of the low-temperature phase V.^11,13,14^ For both materials, the metastable sequence providing the disordered phase II_2_ at a low temperature, a glass transition temperature of 36.9 K, and, coherently, a residual entropy extrapolated at 0 K of 1.48 ± 0.9 J·K^–1^·mol^–1^ ^13^ were found, reinforcing the orientational glassy character of this metastable phase previously determined through X-ray and neutron diffraction.^11,14,15^ With regard to the stable sequence, the low-temperature phase V shows up the greatest difference: For C_4_H_4_S, phase V retains the orientational disorder of phase III, giving rise to an orientational glassy phase V (called V_g_) with a glass transition temperature of 41.7 K,^13^ whereas phase V of C_4_D_4_S was irrefutably found to be a fully ordered phase.^12^

**Figure 1:**
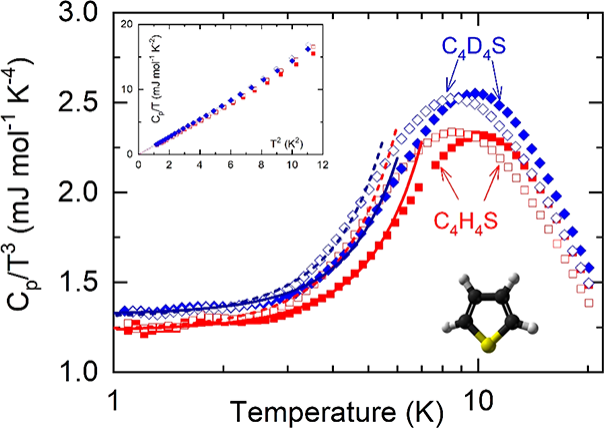
Debye-reduced specific heat data *C*_p_/*T*^3^ for the low-temperature OD metastable (empty symbols) and stable (full symbols) phases of C_4_H_4_S (red squares) and C_4_D_4_S (blue diamonds). Continuous lines are fits corresponding to eq 1. (upper inset) Specific heat data represented as *C*_p_/*T* as a function of *T*^2^ within the very low-temperature range (down to 4 K). (lower inset) Molecule of thiophene: C, black atoms; H or D, gray atoms; and S, yellow atom.

The orientational disorder of the C_4_H_4_S phases was also confirmed through dielectric spectroscopy.^16^ However, experimental thermal conductivity studies on the low-temperature orientational disorder (OD) phases of normal thiophene show a temperature dependence that is typical of ordered crystals.^17,18^

As a summary, the low-temperature phase domain of heterocyclic aromatic molecular crystalline thiophene can display different orientational glasses in which a disorder (or order) level can be operated via a substitution of protons by deuterons. Thus, at a low temperature, two phases can be found for each C_4_H_4_S and C_4_D_4_S material: the metastable (plastic) phase II_2_ and the stable phase V for C_4_H_4_S, both with frozen orientational disorder below the respective glass transitions (at 36.9 and 41.7 K, respectively), and the metastable (plastic) phase II_2_ (exhibiting the same properties as OD phase II_2_ of C_4_H_4_S) and the FO phase V for C_4_D_4_S.

Measurements of the specific heat, *C*_p_, from 1 K up to the melting point were performed for both the metastable and stable phases of C_4_H_4_S and C_4_D_4_S. In particular, for samples C_4_H_4_S (17.2017 mg) and C_4_D_4_S (20.3136 mg) the measurements were carried out with a Quantum Design relaxation-type calorimeter PPMS employing a ^3^He probe in the temperature interval of 1 ≤ *T*, K ≤ 20. In addition, specific heat measurements for the sample C_4_D_4_S (0.31618 g) were performed with a laboratory-made adiabatic calorimeter from 10 to 300 K.^19^ Resolutions of the measurements are ca. 0.3% for the relaxation calorimetry and ca. 0.2% for the adiabatic calorimetry. The results presented next will focus on the low-temperature interval of 1 ≤ *T*, K ≤ 25.

Figure 1 shows the experimental specific heat along with the typical Debye representation, *C*_p_/*T*^3^, expressed as a function of temperature for the metastable (plastic) and stable phases of C_4_H_4_S and C_4_D_4_S. Figure 1 evidences that, independently of whether the phase is OD or FO, all the specific heat curves exhibit a maximum in the *C*_p_/*T*^3^ representation. Despite the similarity of the maximum temperature values, the maximum occurs at slightly lower temperatures for the OD II_2_ phases than the V phases. In addition, deuteration leads to an increase in the *C*_p_ maximum as well as in the Debye contribution to *C*_p_ (*C*_3_), the latter being independent of the ordering character of the phase.

According to its most canonical low-temperature approximation, the specific heat of a crystal can be expressed as a function of temperature as
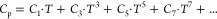
1where the *C*_1_ term accounts for possible “two-level state” (TLS) tunneling effects,^20,21^*C*_3_ accounts for the well-known contribution from acoustic modes (i.e., the Debye model), *C*_5_ accounts for the BP, which can be explained based on different models, and *C*_7_ and additional higher-order terms account for high-temperature features.^22^ Note that this low-temperature approximation applies to temperatures below *T*_max_, at which *C*_p_/*T*^3^ exhibits its maximum.

Within the temperature range of the measurements (*T* ≥ 1 *K*), Figure 1 does not reveal one of the most common and distinctive fingerprints of glasses, the linear term for *C*_p_ at very low temperatures (*C*_1_ in eq 1), for the studied ordered and disordered crystals. To confirm the lack of such a term, we represent *C*_p_/*T* versus *T*^2^ for temperatures lower than 4 K in the inset of Figure 1. On the contrary, our experimental results unambiguously show the existence of a bump (BP) in the glassy crystalline states (orientationally disordered metastable phases, II_2_, for both C_4_H_4_S and C_4_D_4_S) as well as in the disordered stable phase V of C_4_H_4_S. Such a thermal anomaly is a typical feature of OD phases^23^ as well as crystalline phases with occupational disorder, even for weakly bonded molecular crystals.^23−27^ A Boson-like peak also appears in the fully ordered stable low-temperature phase V of C_4_D_4_S with similar intensity to that of phase II,which from the canonical knowledge could be ascribed to the first van Hove singularity. Nevertheless, its characteristic temperature coincides with that of the orientational glassy phase V of C_4_H_4_S (i.e., 10.0 K, see Table 1), and it is also very close to those observed for OD phases II_2_.

**Table 1:** Heat Capacity Coefficients (eq 1) and Characteristic Temperatures^a^

	phase	C_1_	C_3_	C_5_ × 10^3^	C_7_ × 10^5^	*T*_max_	Θ_D_	*T*_g_
material	(state)	mJ K^–2^ mol^–1^	mJ K^–4^ mol^–1^	mJ K^–6^ mol^–1^	mJ K^–8^ mol^–1^	K	K	K
C_4_H_4_S	V(OD)	0 ± 0.05	1.23 ± 0.06	9.8 ± 1	23.7 ± 3	10.0	116.7	41.7
	II_2_(OD)	0 ± 0.05	1.23 ± 0.06	14.6 ± 2	48.0 ± 7	8.4	116.7	36.9
C_4_D_4_S	V(FO)	0 ± 0.05	1.32 ± 0.06	9.9 ± 2	39.7 ± 7	10.0	114.0	
	II_2_(OD)	0 ± 0.05	1.30 ± 0.06	15.0 ± 2	59.9 ± 7	8.5	114.6	39.1

a θ_D_, Debye temperature obtained from *C*_3_ coefficient (θ_D_^3^ = 12π^4^*R*/(5·*C*_3_)); *T*_max_ is the temperature at which *C*_p_/*T*^3^ exhibits the maximum of the specific heat, and glass transition temperature (*T_g_*) for the low-temperature stable (S) and metastable (M) phases of normal and deuterated thiophene. OD is orientational disordered; FO is fully ordered.

For perfectly ordered crystals, like noble gases, the *C*_5_ term accounts for the normal dispersion of the acoustic branches of the crystal,^28^ that is, a kind of forerunner of the bump in normalized heat capacity, which is associated with the first van Hove singularity in the density of vibrational states. Meanwhile, the nature of the *C*_5_ heat capacity term for disordered solids is still under debate, and various theoretical physical interpretations have been proposed for it. Examples include the existence of additional soft anharmonic modes,^29^ the presence of heterogeneities in the elastic constants of the continuous medium,^30−32^ the smearing out of the lowest van Hove singularity appearing in the crystalline counterpart,^33,34^ and the stack of transverse excitations (at frequencies around the BP).^5^

Interestingly, a recent model proposed by Baggioli and Zaccone assembles the explanation of the BP from a unified description regardless of the ordering of the system.^35−37^ This model proposes, as suggested in a recent experimental work,^38^ that the BP in *g*(ω) can simply appear by the piling up of low-energy optical modes provided that their energy is close to that of the acoustic modes at the reciprocal space points of the Brillouin zone boundary. For sufficiently low energies of the first optical mode at the Brillouin zone center (ω_o_), that is, close to the energy of the acoustic modes, the magnitude of the BP is reinforced, whereas for large ω_o_ the contribution to the BP vanishes, regardless the order of the system. Moreover, the model accounts also for the *C*_1_ linear term in eq 1 as due to the damping of the optical modes (that can be also related to anharmonicity for perfectly ordered crystals). For low values of the damping term, *C*_1_ is decreased, whereas the intensity of the BP is enhanced.

It is worth noting that, since there is not a quantum theory for the dynamics of glasses and disordered systems, the theoretical models listed above are mainly phenomenological and provide only a qualitative description of the low-temperature anomalies observed for their specific heat.

The results in Figure 1 (see inset) clearly reveal the nonexistence of the upturn of the curve at the lowest temperatures (∼1 K), which, according to the previous model, would mean that, whatever the phase (ordered or disordered) and compound (normal or deuterated), the damping of the optical modes is negligible. Nevertheless, our first-principles calculations (explained below) show a strong anharmonic character of the ordered phase V of C_4_D_4_S, which suggests that anharmonicity cannot be the only cause for the appearance of the TLS regime (at least not in ordered crystals, as it is predicted by the Baggioli and Zaccone model.^35−37^). Nevertheless, we cannot rule out the possibility that the linear contribution to *C*_p_ appears at temperatures below 1 K.

According to the values of the *C*_3_ coefficient, which is related to the elastic properties of the crystal, the Debye temperatures Θ_D_ of normal and deuterated thiophenes are only slightly different. Specifically, for both the low-temperature phases V and II_2_, Θ_D_(C_4_D_4_S) is less than Θ_D_(C_4_H_4_S) (see Table 1). Because the Debye (elastic) temperature, proportional to the speed of sound, is mainly determined by the speed of transverse acoustic modes (*v*_T_), the small differences between the normal and deuterated Debye temperatures (for both stable phases V and metastable phases II_2_) can be accounted for by the difference in their densities (∼4% according to refs (12) and (14)), which consistently should be accompanied by a similar difference in the shear modulus (*v*_T_^2^ = μ/ρ). Moreover, although the higher molecular weight of deuterated thiophene should lead to a lower temperature for the maximum of *C*_p_/*T*^3^ versus *T*,^38^Figure 1 and Table 1 evidence that the corresponding *T*_max_ values are virtually the same for normal and deuterated samples when comparing the respective phases II_2_ (8.4–8.5 K) and V (10.0 K). Accordingly, the experimental similarity between the excess in the density of vibrational states of the orientational glasses (phases II_2_ of both normal and deuterated, as well as disordered phase V of normal compound) cannot be attributed to the elastic differences (acoustic modes) between different crystalline phases, but univocally to the existence of additional (optical) modes appearing at energies around the BP. This fact is confirmed by first-principles calculations of the density of vibrational states for the well-ordered phase V of C_4_D_4_S using ab initio molecular dynamics (AIMD) simulations based on density functional theory (DFT) (see the Supporting Information for details); we note that, even for the FO phase of deuterated thiophene, standard harmonic DFT approaches cannot provide physically meaningful results due to the inherently high anharmonicity of the system.

In particular, on the one hand, the phonon frequency spectrum calculated with harmonic DFT methods for the ordered phase V of C_4_D_4_S (not shown here) displayed a large number of imaginary phonon modes. On the other hand, AIMD calculations, which fully can take into account the anharmonicity of the system,^39^ rendered a well-behaved density of vibrational states (i.e., all phonon frequencies were real) for the ordered phase V of C_4_D_4_S. Figure 2 shows the calculated *g*(ω) expressed as a function of ω as well as the reduced vibrational density of states *g*(ω)/ω^2^ (inset Figure 2), which clearly evidence the BP for the FO phase.

**Figure 2:**
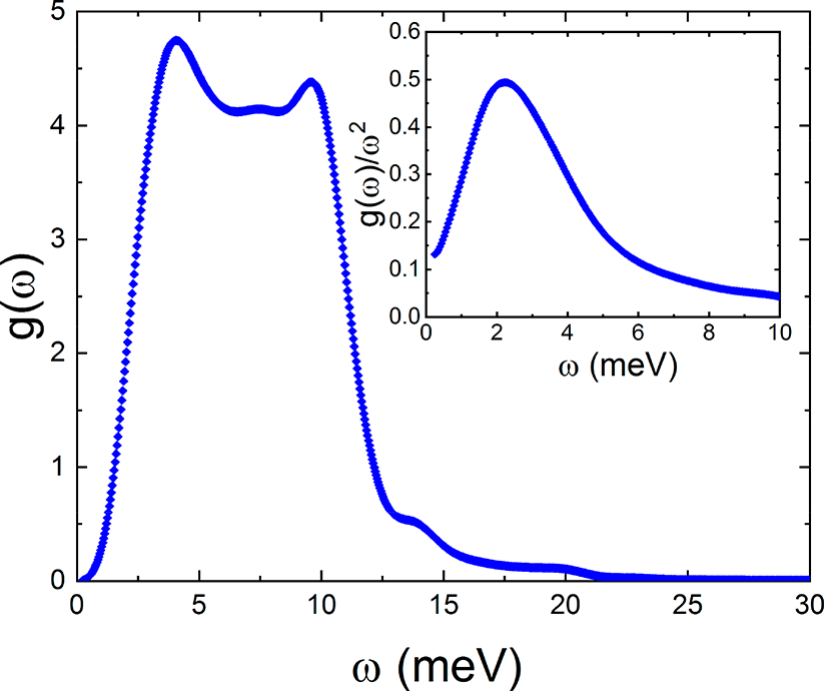
Low-energy part of the vibrational density of state (*g*(ω)) expressed as a function of the energy (ω) for the low-temperature FO phase V of C_4_D_4_S. (inset) Reduced density of states (*g*(ω)/ω^2^ vs ω). Results were calculated theoretically by means of first-principles AIMD approaches based on DFT.

The *C*_v_/*T*^3^ curve that results from the AIMD calculations is qualitatively in good agreement with the experimental observations. In particular, a bump, that is, a BP, appears at low temperatures, and the characteristic signature of the TLS regime is missing. The temperature at which the BP appears in the calculations (∼7 K), however, is slightly lower than the one observed in the experiments (10 K). The quantitative disagreement between our calculations and experiments is reasonable after considering that likely thermal expansion and quantum effects have been neglected in the theoretical results. A comparison between the calculated specific heat (at constant volume *C*_v_) and experimental *C*_p_ values is shown in Figure 3, where the specific heat curves were referred to their Debye values (*C*_3_ coefficient) and normalized.

**Figure 3:**
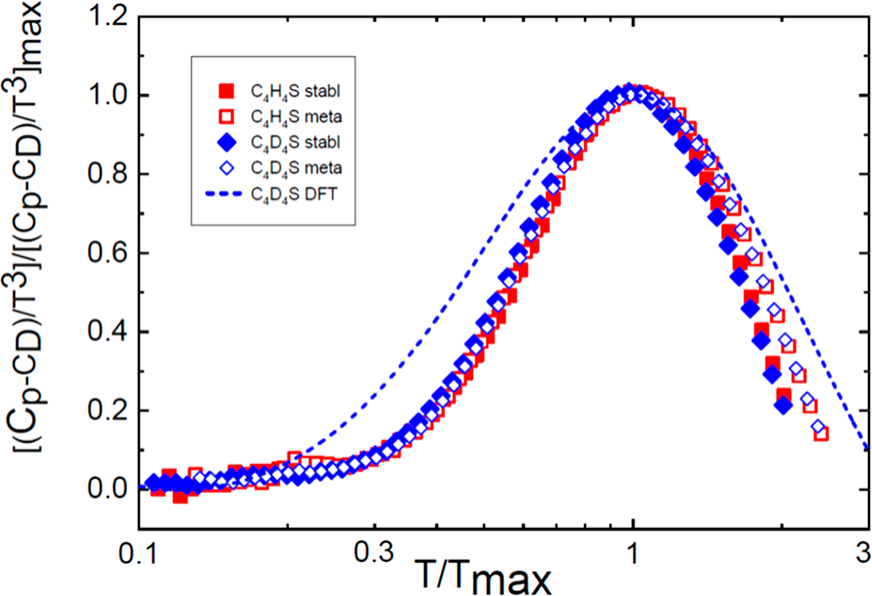
Specific-heat data normalized with respect to Debye values (*C*_D_, i.e., *C*_3_ coefficient for the experimental data) scaled to the height [(*C*_p_ – *C*_D_)/*T*^3^)]/[(*C*_p_ – *C*_D_)/*T*^3^)]_max_ as a function of the temperature normalized to the position of the peak. Dashed line depicts the calculated values (for *C*_v_), whereas symbols (as in Figure 1) concern the experimental values.

From the first derivative of *g*(ω) with respect to ω, we deduce that, in the regime of low vibrational frequencies (ω < 5 meV), the maximum variation of *g*(ω) with respect to ω occurs at ∼2 meV. We tentatively ascribe this energy to the lowest optical phonon branches appearing in C_4_D_4_S (FO phase V), which coincides with the frequency at which the function *g*(ω)/ω^2^ is maximum (inset, Figure 2).

As a consequence, optical phonons within the energy range of strongly dispersive phonon branches indiscernibly contribute to the so-called BP; thus, anomalies in the *C*_p_/*T*^3^ versus *T* representation (or alternatively in *g*(ω)/ω^2^ versus ω) cannot account for the glassy or crystalline character of a given phase. In other words, the so-called BP reflects the features of the crystal dispersion and appears as a consequence of the characteristic acoustic and optical phonon branches of each crystalline phase in such a way that disorder can only modify slightly the density of vibrational states, but it does not seem to be at the origin of the phenomenon.^40^

In addition, the linear term in the heat capacity (not attributed to conduction electrons), repeatedly considered as the fingerprint of glasses, does not seem to be a necessary condition for glasses and, contrary to what has been reported in a recent theoretical study,^32^ it does not seem to be a consequence of the anharmonicity of the system (at least, within the temperature regime analyzed in this work).

To generalize the above conclusion, in Figure 4 we represent the *C*_p_/*T*^3^ function normalized by its Debye contribution and represented as a function of *T*/*T*_max_ for a wide range of solids including atomic (Ar) or diatomic (*p*-H_2_), completely ordered crystals (glycerol, deuterated ethanol (D-ethanol), deuterated thiophene, and cristobalite and coesite SiO_2_), weakly disordered molecular crystals (CCl_4_, CBrCl_3_, CBr_2_Cl_2_), orientational glasses (D-ethanol, normal and deuterated thiophenes, freon112, and freon113), and completely disordered molecular structural glasses (D-ethanol, glycerol, SiO_2_). It is noteworthy that, in the case of simple crystals like Ar and *p*-H_2_, *T*_max_ corresponds to the first feature of the van Hove singularity in the density of vibrational states, which is caused by acoustic vibrational excitations with transverse polarization; in the case of completely ordered crystals, the *T*_max_ corresponds to the low-lying optical branch, and in the case of loose crystals and glasses, the *T*_max_ is the maximum temperature of the so-called BP.

**Figure 4:**
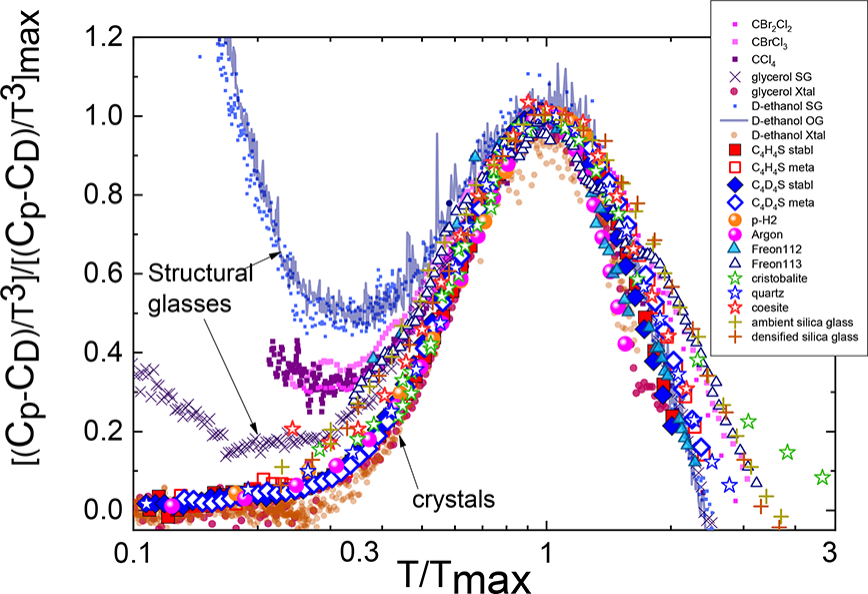
Specific-heat data referred to Debye values (*C*_D_), scaled to the height [(*C*_p_ – *C*_D_)/*T*^3^)]/[(*C*_p_ – *C*_D_)/*T*^3^)]_max_ as a function of the position of the peak for different materials: Low-temperature phase of CCl_4_ (black empty squares), CBrCl_3_ (full red circles), and CBr_2_Cl_2_ (blue empty circles) halomethanes;^38^ structural glasses as glycerol^41^ (pink line), deuterated ethanol (green dots), orientational glass of deuterated ethanol^42^ (continuous green line), freon112 (blue triangles), and freon113 (empty triangles);^24−27^ ordered crystalline phases of glycerol^41^ (pink circles), ethanol^42^ (green circles); stable and metastable phases of normal (full and empty black squares) and deuterated (full and empty black diamonds) of thiophene and well-known materials as *p*-hydrogen^43^ (full orange circles), argon^44^ (full blue circles) and SiO_2_ (glass state as well as the ordered cristobalite and coesite phases).^34^

It is clearly seen that the scaled excess heat capacity of distinct types of solids is characterized by a same trend with universal behavior, both on the left and right of its maximum. A deviation from such a universal behavior appears in some disordered crystal and glasses, when the linear contribution of heat capacity begins to manifest and dominate under a decreasing temperature.^45−50^

We emphasize that the paradigm of the BP will likely find an answer (not provided in this paper) through the study of partially and weakly disordered systems. While structural (canonical) glasses can be described by means of phonon-like optical quasi-localized modes and phonon-like acoustic modes in a pseudo-Brillouin zone, partially and weakly disordered systems with long-range translational order and orientational disorder display genuine acoustic and optical phonons in a real Brillouin zone, which should provide evident clues for the correct answer.

## Supporting Information Available

Stable and metastable phase transitions, discussion of density functional theory calculations, additional references (PDF)

## References

[ref1] Zeller R. C.; Pohl R. O. Thermal conductivity and specific heat of noncrystalline solids. Phys. Rev. B 1971, 4, 2029–2041. 10.1103/PhysRevB.4.2029.

[ref2] Phillips W. A. Amorphous solids: Low temperature properties*;* Springer: Berlin, Germany, 1981.

[ref3] Pohl R. O.; Liu X.; Thompson E. J. Rev. Mod. Phys. 2002, 74, 991–1013. 10.1103/RevModPhys.74.991.

[ref4] Ramos M. A. Are universal “anomalous” properties of glasses at low temperatures truly universal?. Low Temp. Phys. 2020, 46, 104–110. 10.1063/10.0000527.

[ref5] Shintani H.; Tanaka H. Universal link between the boson peak and transverse phonons in glass. Nat. Mater. 2008, 7, 870–877. 10.1038/nmat2293. 18849975

[ref6] Nakayama T. Boson peak and terahertz frequency dynamics of vitreous silica. Rep. Prog. Phys. 2002, 65, 1195–1242. 10.1088/0034-4885/65/8/203.

[ref7] Debenedetti P. G.; Stillinger F. H. Supercooled liquids and the glass transition. Nature 2001, 410, 259–267. 10.1038/35065704. 11258381

[ref8] Cavagna A. Supercooled liquids for pedestrians. Phys. Rep. 2009, 476, 51–126. 10.1016/j.physrep.2009.03.003.

[ref9] Ediger M. D.; Harrowell P. Perspective: Supercooled liquids and glasses. J. Chem. Phys. 2012, 137, 080901 10.1063/1.4747326. 22938210

[ref10] Abrahams S. C.; Lipscomb D. W. N. The crystal structure of thiophene at −55° C. Acta Crystallogr. 1952, 5, 93 10.1107/S0365110X52000198.

[ref11] André D.; Figuiere P.; Fourme R.; Ghelfenstein M.; Labarre D.; Szwarc H. Crystalline thiophene—I: Phase diagram and structures of two orientationally disordered crystalline phases. crystallographic evidence for a metastable low temperature phase. J. Phys. Chem. Solids 1984, 45, 299–309. 10.1016/0022-3697(84)90035-0.

[ref12] Damay F.; Rodríguez-Carvajal J.; André D.; Dunstetter F.; Szwarc H. Orientational ordering in the low-temperature stable phases of deuterated thiophene. Acta Crystallogr., Sect. B: Struct. Sci. 2008, 64, 589–595. 10.1107/S0108768108015103. 18799847

[ref13] Figuiere P.; Szwarc H.; Oguni M.; Suga H. Calorimetric study of thiophene from 13 to 300 K. Emergence of two glassy crystalline states. J. Chem. Thermodyn. 1985, 17, 949–966. 10.1016/0021-9614(85)90008-4.

[ref14] Dunstetter F.; André D.; Gonthier-Vassal A.; Szwarc H.; Ratovelomanana N.; Lautie M.-F. Observation of an incommensurate phase in the stable phase sequence of deuterated thiophene by powder neutron diffraction. Chem. Phys. 1993, 175, 475–482. 10.1016/0301-0104(93)85174-7.

[ref15] André D.; Dworkin A.; Figuiere P.; Fuchs A. H.; Szwarc H. Crystalline thiophene II: A comprehensive study of stable and metastable phases by means of heat capacity, thermally stimulated currents and Raman spectroscopy measurements. J. Phys. Chem. Solids 1985, 46, 505–513. 10.1016/0022-3697(85)90119-2.

[ref16] Pinvidic J.-J.; Takahara S.; Yamamuro O.; Suga H. Dielectric study of crystalline thiophene. Solid State Commun. 1989, 72, 501–505. 10.1016/0038-1098(89)90606-6.

[ref17] Vdovichenko G. A.; Krivchikov A. I.; Korolyuk O. A.; Romantsova O. O. Molecular disorder effects in the thermal conductivity of solid thiophene. Low Temp. Phys. 2014, 40, 1112 10.1063/1.4904001.

[ref18] Korolyuk O. A.; Krivchikov A. I.; Vdovichenko G. A.; Romantsova O. O.; Horbatenko Yu. V. Thermal conductivity of solid thiophene in an incommensurate orientational state. Low Temp. Phys. 2016, 42, 68 10.1063/1.4940993.

[ref19] Kume Y.; Mlyazaki Y.; Matsuo T.; Suga H. Low temperature heat capacities of ammonium hexachlorotellurate and its deuterated analogue. J. Phys. Chem. Solids 1992, 53, 1297 10.1016/0022-3697(92)90249-D.

[ref20] Phillips W. A. Tunneling states in amorphous solids. J. Low Temp. Phys. 1972, 7, 351–360. 10.1007/BF00660072.

[ref21] Anderson P. W.; Halperin B. I.; Varma C. M. Anomalous low-temperature thermal properties of glasses and spin glasses. Philos. Mag. 1972, 25, 1–9. 10.1080/14786437208229210.

[ref22] Pässler R. Limiting Debye temperature behavior following from cryogenic heat capacity data for group-IV, III–V, and II–VI materials. Phys. Status Solidi B 2010, 247, 77–92. 10.1002/pssb.200945158.

[ref23] Ramos M. A.; Vieira S.; Bermejo F. J.; Dawidowski J.; Fischer H. E.; Schober H.; González M. A.; Loong C. K.; Price D. L. Quantitative assessment of the effects of orientational and positional disorder on glassy dynamics. Phys. Rev. Lett. 1997, 78, 82 10.1103/PhysRevLett.78.82.

[ref24] Vdovichenko G.; Krivchikov A.; Korolyuk O.; Tamarit J. Ll.; Pardo L. C.; Rovira-Esteva M.; Bermejo F. J.; Hassaine M.; Ramos M. A. Thermal properties of halogen-ethane glassy crystals: Effects of orientational disorder and the role of internal molecular degrees of freedom. J. Chem. Phys. 2015, 143, 084510 10.1063/1.4929530. 26328859

[ref25] Vispa A.; Romanini M.; Ramos M. A.; Pardo L. C.; Bermejo F. J.; Hassaine M.; Krivchikov A. I.; Taylor J. W.; Tamarit J. Ll. Thermodynamic and kinetic fragility of Freon113: the most fragile plastic crystal. Phys. Rev. Lett. 2017, 118, 105701 10.1103/PhysRevLett.118.105701. 28339247

[ref26] Gebbia J. F.; Ramos M. A.; Szewczyk D.; Jezowski A.; Krivchikov A. I.; Horbatenko Y. V.; Guidi T.; Bermejo F. J.; Tamarit J. Ll. Glassy anomalies in the low-temperature thermal properties of a minimally disordered crystalline solid. Phys. Rev. Lett. 2017, 119, 215506 10.1103/PhysRevLett.119.215506. 29219416

[ref27] Szewczyk D.; Jezowski A.; Vdovichenko G. A.; Krivchikov A. I.; Bermejo F. J.; Tamarit J. Ll.; Pardo L. C.; Taylor J. W. Glassy dynamics versus thermodynamics: the case of 2- adamantanone. J. Phys. Chem. B 2015, 119, 8468–8474. 10.1021/acs.jpcb.5b04240. 26073682

[ref28] Strzhemechny M. A.; Krivchikov A. I.; Jeżowski A. Universal temperature dependence of the thermal conductivity of clathrate compounds, molecular crystals, and glasses at low temperatures. Low Temp. Phys. 2019, 45, 1290–1295. 10.1063/10.0000211.

[ref29] Buchenau U.; Galperin Yu. M.; Gurevich V. L.; Parshin D. A.; Ramos M. A.; Schober H. R. Interaction of soft modes and sound waves in glasses. Phys. Rev. B: Condens. Matter Mater. Phys. 1992, 46, 2798 10.1103/PhysRevB.46.2798. 10003968

[ref30] Klinger M. I.; Kosevich A. M. Soft-mode related origin of the boson peak and acoustic-phonon broadening in glasses. Phys. Lett. A 2001, 280, 365 10.1016/S0375-9601(01)00090-1.

[ref31] Schirmacher W.; Diezemann G.; Ganter C. Harmonic Vibrational Excitations in Disordered Solids and the “Boson Peak. Phys. Rev. Lett. 1998, 81, 136–139. 10.1103/PhysRevLett.81.136.

[ref32] Schirmacher W.; Ruocco G.; Mazzone V. Heterogeneous Viscoelasticity: A combined theory of dynamic and elastic heterogeneity. Phys. Rev. Lett. 2015, 115, 015901 10.1103/PhysRevLett.115.015901. 26182108

[ref33] Chumakov A. I.; Monaco G.; Monaco A.; Crichton W. A.; Bosak A.; Ruffer R.; Meyer A.; Kargl F.; Comez L.; Fioretto D.; et al. Equivalence of the boson peak in glasses to the transverse acoustic van hove singularity in crystals. Phys. Rev. Lett. 2011, 106, 225501 10.1103/PhysRevLett.106.225501. 21702612

[ref34] Chumakov A. I.; Monaco G.; Fontana A.; Bosak A.; Hermann R. P.; Bessas D.; Wehinger B.; Crichton W. A.; Krisch M.; Rüffer R.; et al. Role of disorder in the thermodynamics and atomic dynamics of glasses. Phys. Rev. Lett. 2014, 112, 025502 10.1103/PhysRevLett.112.025502. 24484025

[ref35] Baggioli M.; Zaccone A. Universal origin of boson peak vibrational anomalies in ordered crystals and in amorphous materials. Phys. Rev. Lett. 2019, 122, 145501 10.1103/PhysRevLett.122.145501. 31050477

[ref36] Baggioli M.; Cui B.; Zaccone A. Theory of the phonon spectrum in host-guest crystalline solids with avoided crossing. Phys. Rev. B: Condens. Matter Mater. Phys. 2019, 100, 220201 10.1103/PhysRevB.100.220201.

[ref37] Baggioli M.; Zaccone A. Low-energy optical phonons induce glassy-like vibrational and thermal anomalies in ordered crystals. J. Phys. Mater. 2020, 3, 015004 10.1088/2515-7639/ab4758.

[ref38] Moratalla M.; Gebbia J. F.; Ramos M. A.; Pardo L. C.; Mukhopadhyay S.; Rudic S.; Fernández-Alonso F.; Bermejo F. J.; Tamarit J. Ll. Emergence of glassy features in halomethane crystals. Phys. Rev. B: Condens. Matter Mater. Phys. 2019, 99, 024301 10.1103/PhysRevB.99.024301.

[ref39] Sagotra A. K.; Chu D.; Cazorla C. Influence of lattice dynamics on lithium-ion conductivity: A first-principles study. Phys. Rev. Mater. 2019, 3, 035405 10.1103/PhysRevMaterials.3.035405.

[ref40] Jeżowski A.; Strzhemechny M. A.; Krivchikov A. I.; Davydova N. A.; Szewczyk D.; Stepanian S. G.; Buravtseva L. M.; Romantsova O. O. Heat capacity of molecular solids: The special case of cryocrystals. Phys. Rev. B: Condens. Matter Mater. Phys. 2018, 97, 201201 10.1103/PhysRevB.97.201201.

[ref41] Talón C.; Zou Q. W.; Ramos M. A.; Villar R.; Vieira S. Low-temperature specific heat and thermal conductivity of glycerol. Phys. Rev. B: Condens. Matter Mater. Phys. 2001, 65, 012203 10.1103/PhysRevB.65.012203.

[ref42] Talón C.; Ramos M. A.; Vieira M. A. Low-temperature specific heat of amorphous, orientational glass, and crystal phases of ethanol. Phys. Rev. B: Condens. Matter Mater. Phys. 2002, 66, 012201 10.1103/PhysRevB.66.012201.

[ref43] Krause J. K.; Swenson C. A. Direct measurements of the constant-volume heat-capacity of solid para-hydrogen from 22.79 to 16.19 cm^3^/mol and the resulting equation of state. Phys. Rev. B: Condens. Matter Mater. Phys. 1980, 21, 2533–2548. 10.1103/PhysRevB.21.2533.

[ref44] Finegold L.; Phillips N. E. Low-temperature heat capacities of solid argon and krypton. Phys. Rev. 1969, 177, 1383–1391. 10.1103/PhysRev.177.1383.

[ref45] Ryzhov V. A. Low-energy vibrational excitations in polymethylmetacrylate with IR and RAMAN spectroscopy. Phys. Astron Int. J. 2019, 3, 123–127. 10.15406/paij.2019.03.00169.

[ref46] Johari G. P. Connection between tunneling and localized configurational relaxations in glasses. Phys. Rev. B: Condens. Matter Mater. Phys. 1986, 33, 7201–7204. 10.1103/PhysRevB.33.7201. 9938051

[ref47] Johari G. P. Librational heat capacity of fullerenes in the Einstein model. J. Chem. Phys. 2003, 119, 11912–11916. 10.1063/1.1624052.

[ref48] Johari G. P. Low-energy excitations of guest molecules in clathrates and the Boson peak. Chem. Phys. 2003, 287, 273–283. 10.1016/S0301-0104(02)01006-6.

[ref49] Alexander M. G.; Goshorn D. P.; Onn D. G. Low-temperature specific heat of the graphite intercalation compounds KC_8_, CsC_8_, RbC_8_, and their parent highly oriented pyrolytic graphite. Phys. Rev. B: Condens. Matter Mater. Phys. 1980, 22, 4535–4542. 10.1103/PhysRevB.22.4535.

[ref50] Etrillard J.; Lasjaunias J. C.; Toudic B.; Cailleau H. Low-frequency excitations in incommensurate biphenyl as studied by very low-temperature specific heat. EPL 1997, 38, 347–352. 10.1209/epl/i1997-00250-2.

