# Supplementary material for: Low-Temperature Heat Capacity Anomalies in Ordered and Disordered Phases of Normal and Deuterated Thiophene
Source: J Phys Chem Lett. 2021 Feb 24;12(8):2112–7. doi: 10.1021/acs.jpclett.1c00289 (PMC8594864; doi:10.1021/acs.jpclett.1c00289)
Supplement: Supplementary file 1 — jz1c00289_si_001.pdf [file jz1c00289_si_001.pdf]

Supporting Information

Low-Temperature Heat Capacity Anomalies in Ordered and Disordered Phases of Normal and Deuterated Thiophene

Y. Miyazaki[1], M. Nakano[1], A. I. Krivchikov[2], O. A. Koroyuk[2], J. F. Gebbia[3], C. Cazorla[4], J. Ll. Tamarit[3,*]

[1] Research Center for Thermal and Entropic Science, Graduate School of Science, Osaka University, 1-1 Machikaneyama, Toyonaka, Osaka 560-0043, Japan

[2] B. Verkin Institute for Low Temperature Physics and Engineering of NAS Ukraine, 47 Science Avenue, Kharkov 61103, Ukraine

[3] Grup de Caracterizació de Materials, Departament de Física, EEBE and Barcelona Research Center in Multiscale Science and Engineering, Universitat Politècnica de Catalunya, Eduard Maristany, 10-14, 08019 Barcelona, Catalonia, Spain

[4] Departament de Física, Universitat Politècnica de Catalunya, Campus Nord B4-B5, E-08034 Barcelona, Catalonia, Spain

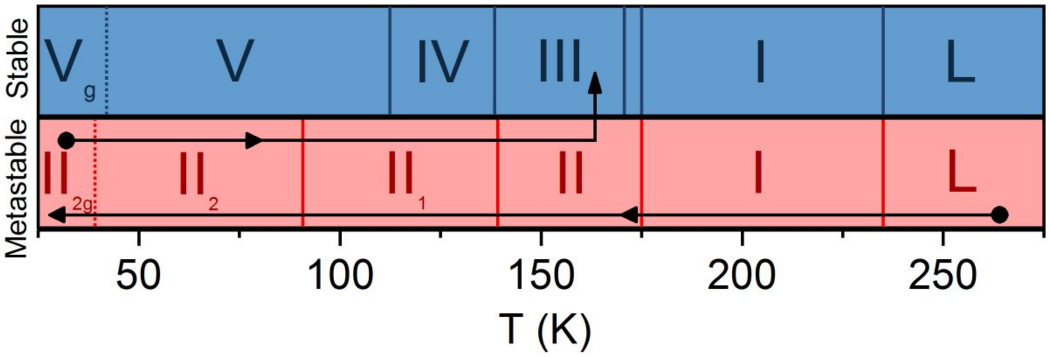

Scheme 1. Stable and metastable phase transitions of normal and deuterated thiophene. Metastable sequence (red) on cooling: L→I→II→II_1→II_2→II_g. On heating: II_g→II_2→II_1→II→I→L. Annealing phase II at ca. 160 K results in the formation of the stable phase III, and consequently to the Stable sequence (blue). Phase V_g is the glass

state of the disordered phase V for normal thiophene, while deuterated thiophene only displays an ordered phase V.

ab initio molecular dynamics simulations based on density functional theory

We performed density functional theory (DFT) calculations based on the PBEsol functional[1] with the VASP software[2]. Long-ranged dispersion interactions in the system were captured with Grimme's DFT-D3 method[3]. Wave functions were represented in a plane-wave basis truncated at 650 eV and a Monkhorst-Pack k-point grid of 4x6x4 was employed for integrations within the Brillouin zone. Geometry relaxations were performed for optimizing the lattice vectors of bulk C_4D_4S (ordered phase V) at zero temperature. To estimate the harmonic density of vibrational states, $g(\omega)$, we employed the small-displacement method[4] and a 2x2x2 supercell containing 576 atoms. To estimate $g(\omega)$ by fully taking into account the anharmonicity of the system, we calculated the Fourier transform of the velocity-velocity autocorrelation function[5] obtained from a long *ab initio* molecular dynamics (AIMD) run (~100 ps) performed at a fixed temperature of 100 K and the equilibrium volume. The supercell employed in the AIMD simulations contained 576 atoms.

(1) Perdew, J.; Ruzsinszky, A.; Csonka, G.; Vydrov, O.; Scuseria, G.; Constantin, L.; Zhou, X.; Burke, K. *Restoring the Density-Gradient Expansion for Exchange in Solids and Surfaces, Phys. Rev. Lett.* **2008**, *100*, 136406.

(2) Kresse G.; Furthmuller, J. *Efficient iterative schemes for ab initio total-energy calculations using a plane-wave basis set, Phys. Rev. B* **1996**, *5*, 11169.

(3) Grimme, S.; Antony, J.; Ehrlich, S.; Krieg, H. *A consistent and accurate ab initio parametrization of density functional dispersion correction (DFT-D) for the 94 elements H-Pu, J. Chem. Phys.* **2010**, *132*, 154104.

(4) Cazorla, C.; Boronat, J. *Simulation and understanding of atomic and molecular quantum crystals, Rev. Mod. Phys.* **2017**, *89*, 035003.

(5) Sagotra, A. K.; Chu, D.; Cazorla, C. *Influence of lattice dynamics on lithium-ion conductivity: A first-principles study. Phys. Rev. Mater.* **2019**, *3*, 035405 (2019)
